# Effects of coenzyme Q10 in a propofol infusion syndrome model of rabbits

**DOI:** 10.2478/abm-2023-0058

**Published:** 2023-10-18

**Authors:** Banu Kilicaslan, Seda B Akinci, Fatma Saricaoglu, Savas O Yılbas, Burcu A Ozkaya

**Affiliations:** Department of Anesthesiology and Intensive Care, Hacettepe University, Ankara 06230, Turkey

**Keywords:** coenzyme Q10, electron, microscopy, mitochondria, propofol infusion syndrome, transmission

## Abstract

**Background:**

Coenzyme Q (CoQ) might be the main site of interaction with propofol on the mitochondrial respiratory chain in the propofol infusion syndrome (PRIS) because of the structural similarity between coenzyme Q10 (CoQ10) and propofol.

**Aim:**

To investigate the effects of CoQ10 on survival and organ injury in a PRIS model in rabbits.

**Methods:**

Sixteen male New Zealand white rabbits were divided into 4 groups: (1) propofol infusion group, (2) propofol infusion and CoQ10, 100 mg/kg was administered intravenously, (3) sevoflurane inhalation was administered, and (4) sevoflurane inhalation and CoQ10, 100 mg/kg intravenously, was administered. Arterial blood gas and biochemical analyses were repeated every 2 h and every 12 h, respectively. Animals that were alive on the 24th hour after anesthesia induction were euthanized. The organ damages were investigated under light and transmission electron microscopy (TEM).

**Results:**

The propofol infusion group had the highest troponin T levels when compared with the other three groups at the 12th hour. The propofol + CoQ10 group had lower troponin T levels when compared with the propofol and sevoflurane groups (*P* < 0.05). Administration of CoQ10 decreased total liver injury scores and total organ injury scores both in the propofol and sevoflurane groups. The propofol and sevoflurane organ toxicities were attenuated with CoQ10 in liver, gallbladder, urinary bladder, and spleen.

**Conclusion:**

The addition of CoQ10 to propofol and sevoflurane anesthesia prevented the propofol-associated increase in troponin T levels at the 12th hour of infusion and decreased anesthetic-induced total liver and organ injury scores.

Propofol is a drug used in anesthesia induction. However, it has also become one of the frequently used drugs in the maintenance of anesthesia and for sedation of critically ill patients in the intensive care unit, because of favorable pharmacokinetics and rapidly reversible sedation effects [1].

Propofol infusion syndrome (PRIS) was reported in 1990 by Bray [2]. According to the most recently updated definition, PRIS occurs in critically ill patients receiving propofol infusion for long periods (>48 h) and high doses (>4 mg/kg/h). Clinical characteristics of PRIS include acute kidney injury (AKI), hyperkalemia, lipidemia, heart failure, fever, elevated liver enzymes, increased lactate levels, metabolic acidosis, rhabdomyolysis, or electrocardiogram changes that cannot be explained otherwise with the presence of one or more clinical conditions [1].

The mechanism causing PRIS has not been clarified, yet. Studies suggest that propofol affects the mitochondrial electron transfer on the respiratory chain. A previous study showed that propofol strongly inhibited complex I activation, with a lower degree of inhibitory effect on complex II + III [3]. In another study using skeletal muscle, propofol has been shown to reduce complex IV activation [4]. When guinea pig heart muscle was exposed to propofol, increases in the oxidation of cytochrome c and cytochrome aa3 were reported [5]. Kam and Cardone [6] speculated that propofol might cause inhibition of coenzyme Q (CoQ) in complex II, cytochrome c in complex IV, or carnitine palmitoyltransferase I and II. There are other studies showing the possible interaction of propofol with fatty acid oxidation. When all these studies are evaluated together, it can be said that the basic pathophysiological mechanism of PRIS is an insufficiency in energy production in the mitochondrial respiratory chain [7].

In a study conducted by Vanlander et al. [8] in rats, propofol has been shown to inhibit electron flow through the respiratory chain, and CoQ was observed to be the main site of interaction with propofol because propofol and coenzyme Q10 (CoQ10) were structurally similar. Moreover, Bergamini et al. [9] reported that cultured cells (T67 and HeLa), pretreated with a CoQ10 formulation, were more resistant to propofol toxicity, confirming the main site of interaction with propofol and the protective effect of CoQ10.

CoQ10 is an essential compound found in the membranes of almost all cells in the human body. It is involved in electron transport chain in mitochondrial membranes during aerobic cellular respiration. A sufficient amount of CoQ10 is required for maintenance of cellular respiration and ATP production [8]. Pretreatment with CoQ10 protected from toxic effects of propofol in human cardiomyocytes [10]. Therefore, CoQ10 could be a potential rescue treatment for PRIS.

In the present study, we aimed to investigate the effect of CoQ10 on survival and organ injury in a PRIS model in rabbits.

## Methods

This project was approved by Hacettepe University Experimental Animals Ethics Committee (Approval Number: 2010/40-4, Date: 01/09/2010), approval being obtained before commencement of any experimental activity. Sixteen healthy male New Zealand white rabbits (3–4 months old, weighing 2500–3500 g) were obtained from the Laboratory Animal Husbandry Facility of Hacettepe University. All animals were kept individually in separate cages under environmentally controlled conditions at 21 ± 2°C and 30%–70% relative humidity with a 12 h dark–12 h light illumination sequence with ad libitum access to tap water. The rabbits were fed with standard laboratory pelleted diet (Laboratory rabbit feed, Optima). The work has been reported in accordance with the Animals in Research: Reporting in Vivo Experiments (ARRIVE) guidelines[11].

An anesthesiologist attended to the animals forming part of the experiment, starting from the premedication till the death of the animals. After fasting all night, the animals were premedicated with intramuscular 5 mg/kg xylazine (Alfazyne 2% Injectable, Alfasan International B. V.) and atropine (Atropine Sulfate, Galen Drug) (0.05 mg/kg). Twenty minutes after premedication, anesthesia was induced with intramuscular 50 mg/kg ketamine (Ketalar Flacon, Pfizer). After anesthesia induction, the animal's trachea was intubated with an endotracheal tube (3.5 mm inner diameter) through tracheostomy and was connected to a mechanical ventilator (Harvard Apparatus, Inspira ASV) with initial settings of respiratory rate of 30 breaths/min, tidal volume of 10 mg/kg, and inspirational oxygen fraction of 100%. An intraarterial catheter was inserted into the central ear artery, and an intravenous cannula (22-gauge polyethylene catheter) was inserted into the marginal ear veins, after which blood was drawn for basal biochemical and metabolic parameters. Arterial blood gas analysis (with the use of GEM Premier 3000, Instrumentation Laboratory; and involving the following parameters: pH, partial pressure of arterial carbon dioxide, partial pressure of oxygen, bicarbonate [HCO_3_], arterial oxygen saturation [SaO_2_], potassium, sodium, calcium, glucose, base excess [BE], hemoglobin, hematocrit, and lactate) was repeated once in every 2 h. Biochemical analyses of sodium (Na^+^), potassium (K^+^), chloride (Cl^−^), blood urea nitrogen (BUN), creatinine (Cr), alanine aminotransferase (ALT), aspartate aminotransferase (AST), lactate dehydrogenase (LDH), gamma-glutamyl transpeptidase (GGT), creatinine kinase (CK), troponin T, total cholesterol (TC), triglycerides, and serum CoQ10 levels were repeated every 12 h. The total blood collected from the rabbits for blood analysis and biochemical tests did not exceed 3–5 mL in 24 h. All the blood samples were sent to Hacettepe University Hospital Clinical Laboratory. For measurement of serum total CoQ10 levels, venous blood was promptly centrifuged at 4°C and the harvested plasma was stored at −80°C until analysis. After extraction with 1-propanol and centrifugation, the supernatant was injected directly into an HPLC system with electrochemical detection as described previously [12].

**Figure 1.**
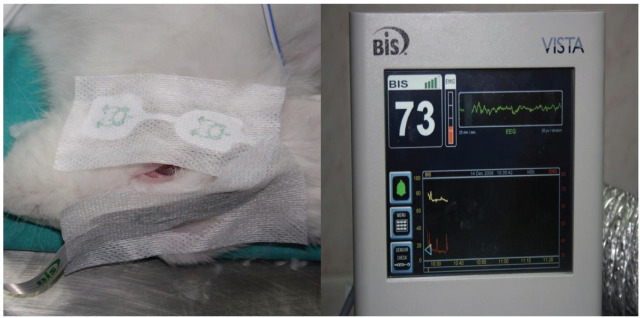
BIS monitoring in the rabbit.

**Figure 2.**
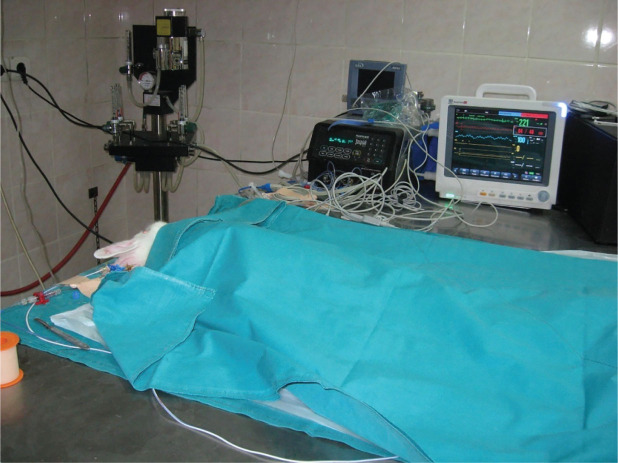
General view of the experimental area.

Routine monitoring during anesthesia included 5-lead ECG, invasive arterial blood pressure monitoring, oxygen saturation measurement with the use of a pulse oximeter (BeneView T5 Patient Monitoring System, Shenzhen Mindray Bio-Medical Electronic CO. LTD.), bispectral index (BIS Vista Monitoring system, Aspect Medical Systems Inc.; **Figure 1**), rectal temperature monitoring, and hourly urinary output after necessary catheterizations (Kendall Curity Foley Catheters, Tyco Healthcare) were inserted. To prevent hypothermia, the animals were actively heated externally (Temperature Management Unit Model 505, Bair Hugger Therapy). The animals were repositioned every 6 h and received subcutaneous 100 IU of enoxaparine (Clexane, Sanofi Aventis) once in every 12 h to prevent thrombosis (**Figure 2**).

After standard premedication and anesthesia induction as described above, the sixteen rabbits were randomly divided (computer based) into 4 groups.

Group 1: Propofol group (n = 4). Propofol infusion (Propofol 2% Fresenius, Fresenius Kabi) was started intravenously with a perfusor (Perfusor Compact, B Braun) at a dose of 20 mg/kg/h. A second perfusor infused 5 mL 0.9% NaCl solution intravenously in 30 min.

Group 2: Propofol + CoQ10 group (n = 4). Propofol infusion was started intravenously with a perfusor (Perfusor Compact, B Braun) at a dose of 20 mg/kg/h. A second perfusor infused CoQ10 (C9538 CoQ10, 1 g, ≥98% [HPLC] Sigma-Aldrich) 100 mg/kg in 5 mL 0.9% NaCl intravenously in 30 min.

Group 3: Sevoflurane group (n = 4). Sevoflurane (Sevorane Likid, Abbott Laboratories) inhalation was started at a dose of 1.5% in 4 L/min oxygen flow. A perfusor infused 5 mL 0.9% NaCl solution intravenously in 30 min.

Group 4: Sevoflurane + CoQ10 (n = 4). Sevoflurane inhalation was started at a dose of 1.5% in 4 L/min oxygen flow. A perfusor infused CoQ10 100 mg/kg in 5 mL 0.9% NaCl intravenously in 30 min.

During the experiments, the depth of anesthesia was titrated via 5 mg/kg/h changes in propofol infusion or 1% change in concentration of sevoflurane inhalation according to group allocation to a target BIS range between 40 and 60. All treatments, other than grouping drugs, were standard in all animals. For analgesia, 5 mg/kg/h ketamine (Ketalar Flacon, Pfizer) infusion was administered to all animals.

At the end of every hour, fluid requirement was meticulously calculated (hourly urine output + blood drawn for laboratory-volume given with drugs) and replaced by 0.9% NaCl solution. Electrolytes were also replaced if indicated according to the laboratory values. Glucose level was kept between 75 mg/dL and 140 mg/dL by either administration of 5% dextrose solution or insulin infusion. In all animals, ventilatory management was adjusted to keep PaCO_2_ between 30 mmHg and 40 mmHg. pH was kept between 7.35 and 7.45 and bicarbonate infusion was given in case of metabolic acidosis (pH <7.25, bicarbonate <15 meq/L). The time of death was recorded by a blinded observer unaware of the groups. Animals that were alive on the 24th hour after anesthesia induction were euthanized with a high dose of ketamine (60 mg/kg).

### Light microscopy (LM) on paraffin sections

The liver, gallbladder, kidney, urinary bladder, spleen, lung, pancreas, striated muscle (quadriceps femoris), and heart were removed immediately after the experimental procedure. Each organ or tissue specimen was divided into two and fixed overnight in 10% neutral buffered formaldehyde or 2.5% buffered glutaraldehid solutions (Sigma-Aldrich) for LM and transmission electron microscopy (TEM) analyses, respectively. Care was taken to obtain the samples from the fixed parts of the organs or tissues each time, i.e. kidney and heart samples were cross-sectioned through their mid-portion. The cortex and medulla containing kidney sections were evaluated. The organ or tissue samples were fixed in 10% phosphate buffered formalin (pH 7.0) at room temperature, rinsed in buffer, and dehydrated in graded series of ethanol before embedding in paraffin by using an automated tissue processor with constant vacuum. Five to seven micrometer thick sections were prepared with a sliding microtome (Leica). Hematoxylin and eosin and Masson trichrome (MT) stained sections (at least 10 sections per specimen) were examined by at least two independent investigators with a Leica DMR microscope. The images were captured via Leica DC500 digital camera and semi-quantitatively evaluated by using Leica application suite (LAS) software. An experienced histologist unaware of the grouping performed the semi-quantitative analysis by grading each parameter between 0 and 3; this procedure was devised as a modification of the one adopted in the study of Akıncı et al. [13]. In brief, four parameters (pulmonary edema, parenchymal congestion, alveolar hemorrhage, and peribronchial–perivascular–interstitial inflammation) were evaluated for the lung (the total maximum score is 12). Five parameters (necrosis, congestion, hepatocellular injury, periportal inflammation, and vacuolar degeneration) were evaluated for the liver (the total maximal score is 15). Two parameters (congestion and fibrosis) were evaluated for the spleen damage (the total maximum score is 6). Two parameters (congestion and necrosis) were evaluated for the kidney damage (the total maximum score is 6). Three parameters (necrosis, congestion, and inflammation) were evaluated for the pancreas, striated muscle, heart, gallbladder, and urinary bladder damage (the total maximum score is 9).

### TEM on plastic sections

The organ or tissue samples were fixed in 2.5% gluteraldehyde in Sorensons’ phosphate buffer (PBS). Following this, they were rinsed in buffer and post-fixed in 1% osmium tetroxide in PBS at 4 °C for 2 h. The samples were dehydrated in graded series of ethanol to absolute ethanol in preparation for embedding in araldite (EMS) with the use of an automated tissue processor. Semi-thin sections were stained with methylene blue – Azur II; thin sections were stained with uranyl acetate and lead citrate, and analyzed on a TEM (Jeol, JEM1400) with an Orius digital camera attached to enable assessment of the organ damage criteria. At TEM level [14, 15], ultrathin sections were qualitatively examined in a blind manner (unaware of the groups). To check the extent of liver injury, the presence of mitochondrial swelling (the width of the hepatocyte mitochondria) was measured on at least 3 pictures, at five points on each picture, and the average values were taken. In total, a minimum of 100 mitochondria were measured for each sample.

### Statistical analysis

Statistical analysis was performed with the SPSS 23.0 program (IBM SPSS Statistics, 581811). The distribution of the data was tested with Shapiro–Wilk tests for normality. Nonparametric data were presented as median values (minimum–maximum) and the Kruskal–Wallis test was used for comparisons between the four groups, while the Mann–Whitney *U* test and the Wilcoxon signed rank test were used for two groups’ comparisons and within group analysis, respectively. The level of significance was accepted as *P* < 0.05 in comparisons of the four groups.

Mean and standard deviation of the different variables (CK 2409 ± 951 IU/L, cholesterol 50 ± 31 mg/dL, myoglobin 26 ± 8.35 ng/mL) increased 3–5 fold after 12 h propofol infusion in the first recreation of the PRIS model in our laboratory [14]. The aim was also to find a decrease in organ injury; therefore, the sample power was based on CPK levels at the 12th hour of sedation, since these levels serve as the main documented pathophysiologically sound finding of organ injury. Sample size was calculated using a statistical power test with a power above 95%, and alpha = 0.008 (Bonferroni correction) was calculated as 3–6 animals per group. Therefore, the 16 animals were planned to be randomized into four groups.

## Results

The amount of fluids given, total urine output, and the total amount of the drugs given, as well as survival data in each group, are detailed in **Table 1**. The total amounts of propofol infused were similar in the propofol and propofol + CoQ10 groups (*P* = 0.486 for two group comparison with Mann–Whitney *U*). Although none of the rabbits in the propofol group but half of the rabbits in the sevoflurane + CoQ10 group survived for 24 h, the differences in the survival data of the four groups did not reach statistical significance (*P* > 0.05) (**Table 1**). The hemodynamic changes were similar in all the four groups; the within group and between group differences in the median arterial blood pressures and heart rates in the four groups did not reach statistical significance (data not shown).The arterial blood gas analysis and serum biochemistry results of the four groups are given in **Table 2**. As only four rabbits survived up to 24 h, only the basal and 12th hour laboratory results have been presented in **Table 2**. The major difference between the four groups was seen in serum troponin T levels at the 12th hour; the propofol infusion group had the highest troponin T levels when compared with the other three groups. The propofol + CoQ10 group had lower troponin T levels when compared with the propofol and sevoflurane groups. The sevoflurane + CoQ10 group also had lower troponin T levels when compared with the propofol and sevoflurane groups (*P* < 0.05). Propofol infusion caused higher trigliceride levels than sevoflurane inhalation; anesthesia and CoQ10 did not have any protective effect on increased trigliceride levels.

**Table 1.** The amount of total fluids and drugs given intravenously, serum CoQ10 levels and the time to death, and survival ratio at the 24th hour after the start of propofol infusion or sevofurane inhalation

	**Propofol**	**Propofol + CoQ10**	**Sevoflurane**	**Sevoflurane + CoQ10**	***P* value**
Total fluid given (mL)	341 (225–875)	186 (100–300)	284 (200–390)	170 (155–200)	0.057
Total urine output (mL)	160 (85–650)	40 (20–100)	150 (50–170)	85 (50–100)	0.073
Total bicarbonate	5 (0–50)	0 (0–10)	0 (0–10)	0 (0–0)	0.424
Total propofol (mg)	1400 (980–2200)	1050 (760–2000)	0 (0–0)	0 (0–0)	NA
Serum CoQ10 level (μg/mL) – basal	0.14 (0.09–0.39)	0.1 (0.06–0.23)	0.26 (0.16–0.56)	0.13 (0.05–0.21)	0.131
Serum CoQ10 level (μg/mL) – 12th hour	0.41 (0.38–0.45)	0.23 (0.12–0.36)	0.28 (0.08–0.58)	0.16 (0.09–0.25)	0.356
Serum CoQ10 level (μg/mL) – 24th hour	N = 0	0.38 (N = 1)	0.24 (N = 1)	0.23 (0.11–0.34)	NA
Time to death (h)	12 (6–20)	18 (16–24)	20 (17–24)	23 (20–24)	0.066
Survival ratio (%) – 24th hour	0 (0)	1 (25)	1 (25)	2 (50)	0.446

Data are given as median (minimum–maximum) or percentage.

CoQ10, coenzyme Q10; N, number of animals alive; NA, not available.

**Table 2.** The laboratory results of the four experimental groups

	**Propofol**	**Propofol + CoQ10**	**Sevoflurane**	**Sevoflurane + CoQ10**	***P* value**
PH – basal	7.37 (7.17–7.64)	7.4 (7.19–7.55)	7.43 (7.17–7.64)	7.35 (7.25–7.5)	0.980
pH – 12th hour	7.40 (7.15–7.47)	7.15 (7.15–7.15)	7.37 (7.28–7.47)	7.4 (7.28–7.46)	0.804
PaCO_2_ – basal (mmHg)	44.5 (14–63)	42 (41–48)	53.5 (14–61)	30 (22–47)	0.481
PaCO_2_ – 12th hour (mmHg)	26 (20–38)	34 (34–34)	25 (24–26)	27 (20–38)	0.595
PaO_2_ – basal (mmHg)	249 (149–371)	258 (214–266)	256 (149–316)	217 (208–251)	0.752
PaO_2_ – 12th hour (mmHg)	240 (56–391)	116 (116–116)	301.5 (228–375)	273 (185–380)	0.508
Ca^+2^ – basal (mmol/L)	1.3 (1–1.75)	1.5 (1.52–1.75)	1.42 (1–1.69)	1.27 (1.07–1.35)	0.237
Ca^+2^ – 12th hour (mmol/L)	1.45 (0.4–1.61)	0.4 (0.4–0.4)	1.34 (1.24–1.45)	1.5 (0.57–1.51)	0.258
Glucose – basal (mg/dL)	299 (135–467)	307 (268–308)	294 (226–463)	320 (135–467)	0.938
Glucose – 12th hour (mg/dL)	111 (47–234)	47 (47–47)	93.5 (76–111)	144 (121–234)	0.162
Lactate – basal (mmol/L)	2.85 (1–10.5)	4 (1.2–10.5)	1.4 (1–3.2)	4.3 (3.9–5.2)	0.190
Lactate – 12th hour (mmol/L)	5 (3.1–8.8)	4.3 (4.3–4.3)	4.05 (3.1–5)	5.3 (3.2–8.8)	0.855
HCO_3_ – basal (mmol/L)	26 (9.6–36.4)	26 (18.3–36)	28 (15.1–34.7)	23.4 (9.6–25.9)	0.548
HCO_3_ – 12th hour (mmol/L)	16.7 (11.8–19.5)	11.8 (11.8–11.8)	16 (14.8–17.2)	16.7 (14.2–17.9)	0.277
BE – basal (mmol/L)	0.35 (−17.6 to 13.5)	1.2 (−9.9 to 13.5)	3.6 (−6.2 to 11.1)	0.2 (−17.6 to 0.3)	0.586
BE – 12th hour (mmol/L)	−7.2 (−17.1 to 14.2)	−17.1 (−17.1 to 17.1)	−10.1 (−13 to 7.2)	8.8 (−8.1 to 14.2)	0.294
Hematocrit – basal (%)	34 (30–38)	41 (35–46)	37 (35–48)	37 (35–40)	0.282
Hematocrit – 12th hour (%)	34 (34–34)	41 (38–45)	29 (22–43)	35 (33–42)	0.141
ALT – basal (U/L)	32 (26–141)	59 (44–68)	63 (33–163)	95 (62–110)	0.484
ALT – 12th hour (U/L)	84 (29–254)	86 (63–214)	252 (37–440)	112 (93–487)	0.606
AST – basal (U/L)	86 (10–518)	38 (28–41)	46 (25–108)	53 (30–74)	0.606
AST – 12th hour (U/L)	202 (100–1166)	106 (90–820)	509 (107–1421)	98 (89–627)	0.409
GGT – basal (U/L)	9 (7–21)	12 (8–18)	8 (6–21)	12 (9–15)	0.628
GGT – 12th hour (U/L)	9 (4–16)	10 (7–102)	9 (7–16)	16 (9–17)	0.676
Creatinine – basal (mg/dL)	1.08 (0.9–1.20)	1.12 (1.1–1.16)	1.01 (0.8–1.15)	0.9 (0.9–1.02)	0.204
Creatinine – 12th hour (mg/dL)	1.6 (0.8–3)	2.14 (1.35–4.33)	1.2 (0.9–3.2)	1.4 (1.2–2.14)	0.631
LDH – basal (U/L)	746 (688–900)	932 (444–1540)	689 (600–720)	629 (362–1410)	0.508
LDH – 12th hour (U/L)	1356 (1287–2200)	1654 (1500–1944)	1891 (1748–2595)	845 (814–877)	0.078
CK – basal hour (U/L)	1847 (902–2137)	2922 (2545–3300)	1683 (1436–2089)	1724 (1305–2203)	0.191
CK – 12th hour (U/L)	3838 (2961–5876)	3118 (2900–5402)	4236 (3594–9921)	11415 (6000–12769)	0.117
Amylase – basal (U/L)	289 (281–350)	285 (246–356)	286 (218–333)	291 (231–310)	0.820
Amylase – 12th hour (U/L)	485 (440–625)	371 (323–605)	341 (291–510)	449 (364–1084)	0.289
Troponin T – basal (ng/mL)	0.03 (0.01–0.8)	0.005 (0–0.001)	0.05 (0.01–0.18)	0.01 (0–0.01)	0.535
Troponin T – 12th hour (ng/mL)	0.94 (0.2–3.5)	0.005 (0–0.026)*#	0.15 (0.13–0.2)*	0.01 (0–0.01)*#	***0.007***
Cholesterol – basal (mg/dL)	49 (32–55)	30 (27–57)	47 (27–92)	32 (29–82)	0.795
Cholesterol – 12th hour (mg/dL)	119 (64–134)	119 (43–124)	49 (24–81)	48 (29–68)	0.138
Triglicerides – basal (mg/dL)	156 (62–201)	168 (67–270)	60 (43–84)	92 (98–152)	0.139
Triglicerides – 12th hour (mg/dL)	993 (663–5460)	1013 (213–1549)#	106 (62–270)*	325 (126–525)	***0.038***

Data are given as median (minimum–maximum).

* *P* < 0.05 when compared with the propofol group. (*P* = 0.07: the propofol infusion group had the highest troponin T levels when compared with the other three groups.)

# *P* < 0.05 when compared with the sevoflurane group. (*P* = 0.038: Propofol infusion caused higher trigliceride levels than sevoflurane inhalation; anesthesia and CoQ10 did not have any protective effect on increased trigliceride levels.)

ALT, alanine aminotransferase; AST, aspartate aminotransferase; BE, base excess; Ca, calcium; CK, creatinine kinase; CoQ10, coenzyme Q10; GGT, gamma-glutamyl transpeptidase; HCO_3_, bicarbonate; LDH, lactate dehydrogenase; PaCO_2_, partial arterial carbon dioxide pressure; PaO_2_, partial arterial oxygen pressure.

As presented in **Table 3**, group-blinded organ-specific histopathological grades and total scores were calculated for each group. Administration of CoQ10 decreased total liver injury scores and total organ injury scores when compared with the equal volume normal saline administration, in the cases of additions both to propofol infusion and sevoflurane inhalation (*P* < 0.05).

**Table 3.** The histopathologic organ injury scores

	**Propofol**	**Propofol + CoQ10**	**Sevoflurane**	**Sevoflurane + CoQ10**	***P* value**
**Lung**					
Pulmonary edema	2.5 (2–3)	2.5 (1–3)	2.5 (2–3)	2.5 (2–3)	0.983
Parenchymal congestion	3 (2–3)	2.5 (2–3)	2.5 (2–3)	2.5 (2–3)	0.870
Alveolar hemorrhage	1 (1–2)	1 (1–3)	1 (1–2)	1 (1–1)	0.794
Inflammation	2 (2–2)	2 (1–3)	2 (2–2)	2 (2–2)	1
Lung injury score	8.5 (7–10)	8.5 (5–10)	8.5 (7–9)	8 (7–9)	0.926
**Liver**					
Necrosis	0.5 (0–1)	0 (0–0)	1 (0–1)	0 (0–0)	0.061
Hepatocellular injury	2 (2–3)	1 (1–2)	2 (2–2)	1 (1–2)	***0.028***
Congestion	2.5 (2–3)	2 (1–2)	2.5 (2–3)	1 (1–2)	***0.038***
Inflammation	1.5 (1–2)	1 (0–1)	2 (1–2)	0.5 (0–1)	***0.039***
Vacuolar degeneration	3 (2–3)	1.5 (1–2)	2 (2–3)	2 (2–2)	***0.037***
Liver injury score	10 (7–11)	5 (4–7)*#	9 (7–11)	4.5 (4–7)*#	***0.016***
**Spleen**					
Congestion	1.5 (1–2)	0.5 (0–1)	1 (1–2)	0.5 (0–1)	0.075
Fibrosis	0 (0–1)	0 (0–0)	0 (0–1)	0 (0–0)	0.543
Spleen injury score	1.5 (1–3)	0.5 (0–1)	1 (1–3)	0.5 (0–1)	0.079
**Kidney**					
Congestion	1.5 (1–2)	1 (1–1)	1 (1–2)	1 (1–2)	0.475
Necrosis	2 (1–3)	1 (1–2)	1.5 (1–3)	2 (2–2)	0.306
Kidney injury score	3.5 (2–5)	2 (2–3)	2.5 (2–5)	3 (3–4)	0.257
**Heart**					
Congestion	1 (0–1)	0 (0–1)	0.5 (0–1)	0 (0–1)	0.454
Necrosis	0 (0–0)	0 (0–0)	0 (0–0)	0 (0–0)	1
Inflammation	0 (0–1)	0 (0––0)	0.5 (0–1)	0 (0–0)	0.238
Heart injury score	1 (0–2)	0 (0–1)	1 (0–2)	0 (0–0)	0.177
**Muscle**					
Congestion	1 (0–1)	0 (0–1)	0.5 (0–1)	0 (0–0)	0.172
Necrosis	0 (0–0)	0 (0–0)	0 (0–0)	0 (0–0)	1
Inflammation	0 (0–1)	0 (0–0)	0 (0–0)	0 (0–0)	0.392
Muscle injury score	1 (0–2)	0 (0–1)	0.5 (0–1)	0 (0–0)	0.146
**Gall bladder**					
Congestion	1 (0–1)	1 (0–1)	1 (1–2)	1 (0–1)	0.419
Necrosis	2 (0–2)	0.5 (0–1)	2 (1–2)	1 (0–1)	***0.023***

Data are presented as median (minimum–maximum).

(Administration of CoQ10, except the necrosis score, decreased total liver injury scores, total organ injury scores when compared with the equal volume normal saline administration, in the cases of additions both to propofol infusion and sevoflurane inhalation)

* *P* < 0.05 when compared with the propofol group.

# *P* < 0.05 when compared with the sevoflurane group.

CoQ10, coenzyme Q10.

**Figure 3.**
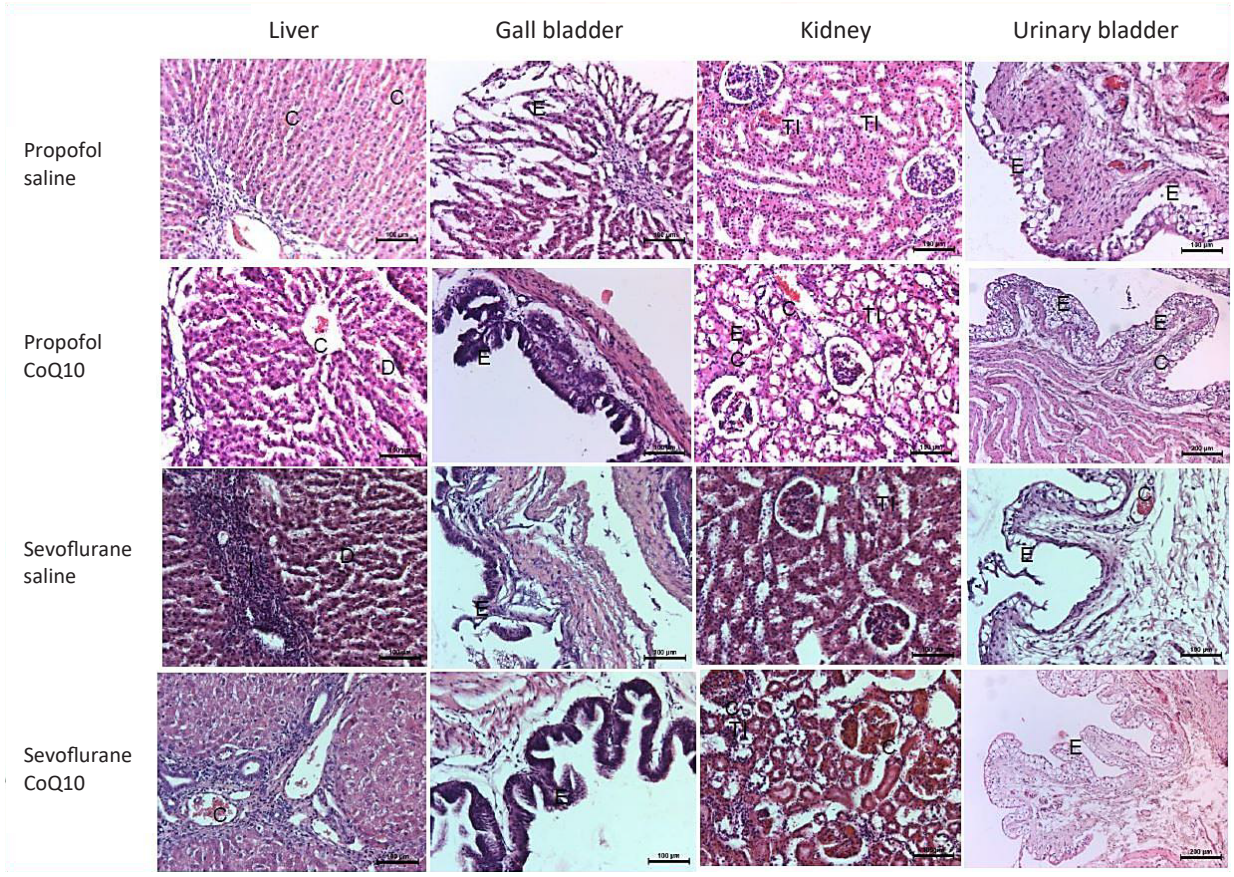
Light micrographs of the liver, gall bladder, kidney, and urinary bladder after the propofol or sevoflurane exposure with respect to saline or additional CoQ10 therapy (hematoxylin and eosin; 200×). The propofol and sevoflurane organ toxicities were recovered up to different extents with CoQ10 therapy. The desquamated mucosal epithelia recovered in the gall bladder and the urinary bladder with CoQ10 (the second and fourth column light micrographs). The portal inflammation, hepatocytic vacuolation, sinusoidal congestion, and dilatation partially improved with CoQ10 treatment (the first column). The kidney damage, which consisted of mainly vascular congestion and tubular injury, did not change much with the CoQ10 treatment (third column). C, vascular congestion; CoQ10, coenzyme Q10; D, dilatation; E, epithelium; I, inflammation; TI, tubular injury.

**Figure 4.**
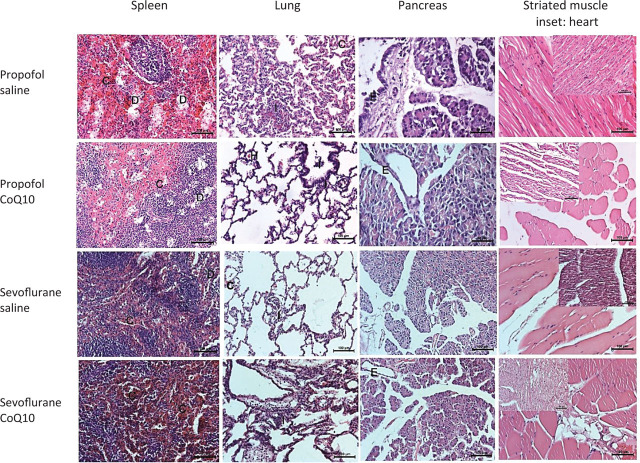
Light micrographs of the spleen, lung, pancreas, and striated muscle (the heart) inset after the propofol or sevoflurane exposure with respect to saline or additional CoQ10 therapy (hematoxylin and eosin; 200×).

**Figures 3** and **4** show the histologic examination of the specimens from the four groups. The propofol and sevoflurane organ toxicities were recovered up to different extents with CoQ10 application in several organs (liver, gall bladder, urinary bladder, and spleen). The histopathology scores remained almost the same in the lungs and kidneys with and without CoQ10. The pancreas, heart, and the striated muscle samples were significantly affected by none of the anesthetic agents in this experimental model. The sevoflurane and propofol toxicity involved the mucosal layers of both the gallbladder and the urinary bladder. The desquamated mucosal epithelia recovered in the gallbladder and the urinary bladder with CoQ10 (**Figure 3**). The portal inflammation, hepatocytic vacuolation, sinusoidal congestion, and dilatation partially improved with CoQ10 treatment (**Figure 3**). The kidney damage mainly consisted of vascular congestion and tubular injury. The proximal tubules were more prone to damage than distal tubules in both groups. The principal region of tubular necrosis in the propofol group was in the corticomedullary junction, in contrast with the sevoflurane group, which exhibited damage in both the medulla and cortex. At the TEM level, tubular cell swelling, nuclear loss, tubular simplification, and brush border loss were apparent in all groups. CoQ10 treatment did not significantly change the propofol- and sevuflorane-induced tubular damage (**Figure 3**). The sevoflurane- and propofol-induced splenic venous sinusoidal congestion and dilatation recovered partially with CoQ10 (**Figure 4**). In neither of the two anesthetic-applied groups was there any significant change, pursuant to the infusion of CoQ10, in the mild to moderate pulmonary alveolar and bronchiolar inflammation, edema, congestion, or hemorrhage. The pancreas, striated muscle, and the heart samples were almost intact despite a few minor findings in all groups (**Figure 4**).

The propofol and sevoflurane organ toxicities were recovered up to different extents with CoQ10 therapy. The splenic venous sinusoidal congestion and dilatation recovered partially with CoQ10 in both drugs (the first column micrographs). The mild to moderate alveolar and bronchiolar inflammation and hemorrhage did not significantly change with CoQ10 in both groups (the second column). The pancreas, striated muscle, and the heart samples were almost intact in all groups. C, vascular congestion; CoQ10, coenzyme Q10; D, dilatation; E, epithelium; H, hemorrhage; I, inflammation.

A mild portal inflammation, mild to moderate sinusoidal congestion with the dilation of centrolobular vein and the sinusoids, and focal hepatocyte degeneration mainly consisting of cell swelling and vacuolation were observed in the sevuforane- and propofol-applied liver specimens. At the TEM level, the propofol- and sevoflurane-applied groups exhibited different ranges of mitochondrial swelling comparing to that of the CoQ10-treated groups. Although the mitochondrial membrane was generally intact in all groups, loss of cristae and a decrease in matrix density were prominent in anesthetic-applied groups. The lesser mitochondrial diameter measurement was significantly increased in the sevoflurane- and propofol-applied groups in comparison with the CoQ10-treated groups. The liver damage partially recovered with CoQ10 treatment (**Figure 4**).

## Discussion

In this study, we investigated the effect of CoQ10 on propofol-infusion–induced organ injury and survival in rabbits. We found that addition of CoQ10 prevented the propofol-associated increase in troponin T levels at the 12th hour of infusion. CoQ10 decreased total liver injury and total organ injury scores when added to propofol infusion and sevoflurane anesthesia. Electron microscopy revealed CoQ10's reversal of anesthetic-associated intramitochondrial structural changes.

Because CoQ10 is present in cell membranes and mitochondria as an energy transfer molecule, and its levels are high in organs with high metabolism such as the heart and the liver [15, 16], its protective effects were more prominent in our study, especially on propofol-associated heart injury and anesthetic-associated liver injury. The cardioprotective effects of CoQ10 have been previously documented against hyperlipidemia-induced cardiac damage in apolipoprotein E-deficient mice, and doxorubicin-induced cardiotoxicity in rats [17, 18]. The clinical evidence for CoQ10 supplementation in heart failure has documented functional improvement in humans with no adverse hemodynamic profile or safety issues [19]. CoQ10 supplementation might be useful as an adjuvant in the treatment of not only heart failure but also many other cardiovascular diseases, such as atrial fibrillation, myocardial infarction, hypertension, insulin resistance, dyslipidemias, and obesity [16].

Similar to our findings, CoQ10 exerted a hepatoprotective effect in a fructose-induced fatty liver model in rats, in high-fat, atherogenic, diets associated liver pathologies [20, 21]. In previous studies on PRIS syndrome in rabbits, propofol infusion caused increased levels of lipids compared with sevoflurane inhalation [14, 22]. It has previously been suggested that lipid accumulation may cause oxidative stress in the liver [21]. The CoQ10 treatment of rabbits that were fed on a high-fat diet reduced the lipid concentrations in liver mitochondria with no effect on plasma lipids, restored mitochondrial CoQ10, and reduced mitochondrial reactive oxygen species levels [21]. CoQ10 did not have any protective effect on propofol-associated hypertriglyceridemia levels, in our study. Even 12 week CoQ10 supplementation in humans did not have any effect on TC, or triglycerides [23] in one study, while much longer-term CoQ10 supplementation significantly reduced serum triglyce-rides levels in other studies [24].

As it has a central role in energy and lipid metabolism, CoQ10 plays a key role in metabolism of all organs including but not limited to the heart and the liver. In our study, we demonstrated that CoQ10 decreased anesthetic-associated total organ injury scores, as evaluated with the use of histological examination. In another study modeling PRIS syndrome in rabbits, continuous infusion of propofol for the sedation for prolonged mechanical ventilation induced fatal multiorgan dysfunction syndrome, in which histologic examination revealed myocarditis, pulmonary edema, hepatitis, and steatosis, as well as focal liver necrosis, cholangitis, gallbladder necrosis, acute tubular necrosis of the kidneys, focal loss of the urinary bladder epithelium, and rhabdomyolysis of skeletal muscles [22].

Rabbits share many clinical signs of PRIS observed in humans. In our study, the presentation of PRIS included lipemic plasma, metabolic acidosis, elevated troponin T, liver enzymes, hypertriglyceridemia, rhabdomyolysis (elevated CK), and myoglobinuria; and finally it was characterized by acute bradycardia progressing to asystole. Sedation with sevoflurane is safe in long-term mechanical ventilation. To distinguish the effects of propofol sedation from those of prolonged mechanical ventilation, the animals were sedated by an alternative sedative inhalation anesthetics drug, namely sevoflurane, as in previous studies in rabbits [22].

In studies examining the effects of propofol infusion on organs, the analysis of biochemical data is usually done at 3 h or 6 h intervals [25, 26]. It has been reported that the significant differences found in most of these parameters were first observed in the vehicle group, 6 h after the onset of infusion, whereas with propofol, some parameters, which were highly sensitive to propofol treatment, increased only after 12 h (CK) or 18 h of the infusion (ALT and AST) [25]. We performed the biochemical analysis every 12 h and the blood gas analysis every 2 h, like Ypsilantis et al. [22]. It may be useful to monitor biochemical parameters at 3 h or 6 h intervals to detect the effects of propofol infusion at the earliest stage. However, another point to be considered in animal studies is that blood sampling from animals should not exceed a certain volume since blood volumes are limited [27].

There is currently no consensus regarding the dose of CoQ10 that should be used in rabbits. In a study in rats, 10 mg/kg and 100 mg/kg doses of CoQ10 were administered [28]. There are certain studies in which CoQ10 is administered to mice at a dose of 10 mg/kg [29]. In another study evaluating the antioxidant and healing effects of CoQ10 in mice, the Q10 dose was administered as 100 mg/kg [30]. Inspired by these studies, we used CoQ10 at a dose of 100 mg/kg in rabbits to obtain antioxidant and healing effects.

Generally, plasma CoQ10 levels are used to determine the tissue CoQ10 status of an organism. This level was determined as 0.40–1.91 μmol/L (0.34–1.65 μg/mL) [31]. However, these levels reflect dietary intake rather than tissue status. The relationships between plasma, tissue, and, most importantly, mitochondrial CoQ10 levels are not clear yet [32]. Previous studies showed that the nutritional supplementation of CoQ10 did not increase its levels in issues above the normal once these tissue levels had attained saturation with CoQ10 [33]. The median blood CoQ10 levels in our study were similar to the mean blood levels of CoQ10 in rabbits (0.29 ± 0.07 μg/mL) and pediatric patients in other studies [34, 35]. We could not detect a rise in serum CoQ10 levels after its administration. Bergamini et al. [9] reported that CoQ10 treated cells used more CoQ10 and increased O_2_ consumption as well as the ATP/ADP ratio. For this reason, we thought that coenzyme was rapidly transported to mitochondria and consumed there, although we could not measure intracellular or intramitochondrial levels to prove this speculation in our study. Actually, plasma or serum CoQ10 concentrations are usually employed for the assessment of CoQ10 status in humans primarily because of the ease of sampling. Although plasma CoQ10 concentrations may not necessarily reflect tissue status [32, 33], the clinical monitoring of plasma CoQ10 concentration provides valuable information in the follow up of treatment of degenerative neurologic and muscular diseases [36]. Lymphocyte and platelet CoQ10 concentrations can also be considered as potential proxies for tissue CoQ10 status [31]. Although Turunen et al. [37] revealed that increased tissue levels after CoQ administration were found in CoQ-deficient patients, suggesting that tissues’ CoQ uptake entails CoQ deficiency, not much work has been done in this area, possibly due to the additional steps required in sample preparation and processing. Assays of the activities of CoQ10-dependent enzyme systems in the respiratory chain in biopsy tissue samples are sometimes performed in several laboratories [31]. In one study, it was found that CoQ10 levels were increased in the plasma of all supplemented subjects, while a corresponding increase was found only in the muscles [38].

Our study also has limitations. First, we chose a high CoQ10 dose and presented its protective effects but we definitely do not know the optimal dose of CoQ10. Second, rabbit metabolism may not directly reflect human metabolism of anesthetics, as always discussed in all animal experiments. Third, although CoQ10 has been used for the treatment of several pathologies, the uptake mechanism of CoQ10 from plasma to tissues is still largely unknown. As discussed above, the plasmatic level of CoQ10 cannot be used as an indicator of tissues’ CoQ10 implementation. We could not detect a rise in serum CoQ10 levels after its administration, and we could measure the CoQ10 level neither in the muscle tissue nor at the mitochondrial level. The fourth is that, unfortunately, we could not include the photos of the swollen mitochondria of the animals in whom CoQ10 was administered, as procured by the use of the TEM, because we could not obtain them due to technical problems. Finally, we believe and accept that the low number of animals in each group resulted in a high variability of the results and limited the more widespread protective effects of CoQ10 reaching statistical significance.

## Conclusions

Administration of CoQ10 protects from propofol-associated cardiac injury, and anesthetic-associated liver injury and total organ injury, in rabbits. We believe this study may prompt studies of CoQ10 treatment for PRIS syndrome that involve human participants.
